# Alphabet Handwriting Recognition: From Wood‐Framed Hydrogel Arrays Design to Machine Learning Decoding

**DOI:** 10.1002/advs.202404437

**Published:** 2024-11-04

**Authors:** Guihua Yan, Xichen Hu, Ziyue Miao, Yongde Liu, Xianhai Zeng, Lu Lin, Olli Ikkala, Bo Peng

**Affiliations:** ^1^ College of Environmental Engineering Henan University of Technology Zhengzhou 450001 China; ^2^ Department of Applied Physics Aalto University Aalto FI‐00076 Finland; ^3^ College of Energy Xiamen University Xiamen 361102 China; ^4^ Department of Materials Science Advanced Coatings Research Center of Ministry of Education of China Fudan University Shanghai 200433 China

**Keywords:** handwriting recognition, hydrogel, machine learning, wood

## Abstract

Handwriting recognition is a highly integrated system, demanding hardware to collect handwriting signals and software to deal with input data. Nonetheless, the design of such a system from scratch with sustainable materials and an easily accessible computing network presents significant challenges. In pursuit of this goal, a flexible, and electrically conductive wood‐derived hydrogel array is developed as a handwriting input panel, enabling recognizing alphabet handwriting assisted by machine learning technique. For this, lignin extraction‐refill, polypyrrole coating, and polyacrylic acid filling, endowing flexibility, and electrical conduction to wood are sequentially implemented. Subsequently, these woods are manufactured into a 5 × 5 array, creating a matrix of signals upon handwriting. Efficient handwritten recognition is then achieved through appropriate manual feature extraction and algorithms with low complexity within a computing network, as demonstrated in this work, the strategic choice of expertise‐based feature engineering and simplified algorithms effectively boost the overall model performance on handwriting recognition. With potential adaptability, further applications in customized wearable devices and hands‐on healthcare appliances are envisioned.

## Graphical abstract

A flexible and electrically conductive wood‐derived hydrogel array is designed as a handwriting input panel, enabling alphabet handwriting recognition assisted by machine learning technique. This work promotes sustainable manufacture in customized wearable devices and hands‐on healthcare appliances.

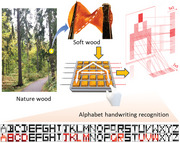

## 1 Introduction

Handwriting recognition is a process, in which the handwritten input from the touching panel is handled and identified into plausible letters, words, and characters. It generically requires the hardware, i.e., a touching pad to collect writing signals, and the software that enables recognition.^[^ ^1^ ^]^ Marveling at sophisticated commercial handwriting recognition systems, which embrace the highly integrated electronic complex and advanced computer algorithms to discern handwritten inputs, whereas realizing handwriting recognition even in the simplest manner with easily available materials and algorithms remains grandly challenged.^[^ ^2^ ^]^

Apart from commercial touching pads made of plastic and metallic complexes, the demand to develop new types of touching pads that allow the collection of handwriting signals is immense.^[^ ^2^, ^3^ ^]^ Specifically, from the viewpoints of environmental protection, bio‐based materials promise tremendous potentials in constituting electronic devices.^[^ ^4^ ^]^ Recently, wood‐derived materials have gained a surge of attention, given their abundance, sustainability, and biodegradability.^[^ ^5^ ^]^ The pristine woods are inherently electrically insulative, while the writing input unit needs to be electrically conductive and can vary its local electrical property upon pressing.^[^ ^5^, ^6^ ^]^ The additional features, e.g., flexibility, are favorable in catering to the practical demand of bioelectronics as haptic sensors.^[^ ^3^, ^4^ ^]^ Prior attempts in preparing conductive wood‐based devices have shown success through surface decoration with metal nanowires, surface carbonization, and polymer filling.^[^ ^7^ ^]^ Nevertheless, the reduced mechanical performance and inherent rigidity limit their further application in writing input appliances. In addition, an array of touching pixels is required in the design of input panels for sufficient recognition resolution.^[^ ^3^ ^]^

As technology continues to advance, seeking free alternatives to costly commercial algorithms become increasingly important to make handwriting recognition more widely accessible to the public. With the rapid growth of machine learning (ML) in recent years, driven largely by its potent computing capability and effectiveness to deal with large databases, it has received irresistible attention from all fields.^[^ ^8^ ^]^ The conceptual “Machine Learning” refers to the learning process inspired by biological cognition.^[^ ^9^ ^]^ Particularly, the working principle of artificial neural network (ANN), a branch of machine learning, resembles that of biological neurons, which highly depends on a feedback system, allowing for self‐correcting.^[^ ^10^ ^]^ Regarding recent efforts in the application of ANN in various pressure sensors,^[^ ^11^ ^]^ it might be the promising algorithmic support for handwriting recognition.^[^ ^12^ ^]^

Here, we report a prototype design of wood‐derived hydrogel arrays to collect the writing tracks, integrated with the readily accessible ML platform in TensorFlow,^[^ ^13^ ^]^ realizing alphabet recognition with accuracy and efficacy. First, we demonstrate an adapted extraction‐refill strategy to render wood flexibility and electrical conduction^[^ ^14^ ^]^ through sequential removal and refilling of lignin, polypyrrole coating, and polyacrylic acid infusion. Next, we devise this wood‐framed hydrogel into a 5 × 5 grid of a circuit and evaluate its capacity to capture handwritten patterns, which serve as the input for our ML models. Ultimately, after implementing appropriate feature extraction on data processing and algorithm selection, we achieve high‐quality classification results across different user inputs, showcasing the practical value of our ecofriendly, cost‐effective, and portable solution for handwritten language recognition and translation. The combination of wood‐framed hydrogel arrays and ML will prompt the development of a sustainable miniature vocabulary recognition system.

## 2 Results

### 2.1 Design and Characterization of Soft Wood

**Figure 1:**
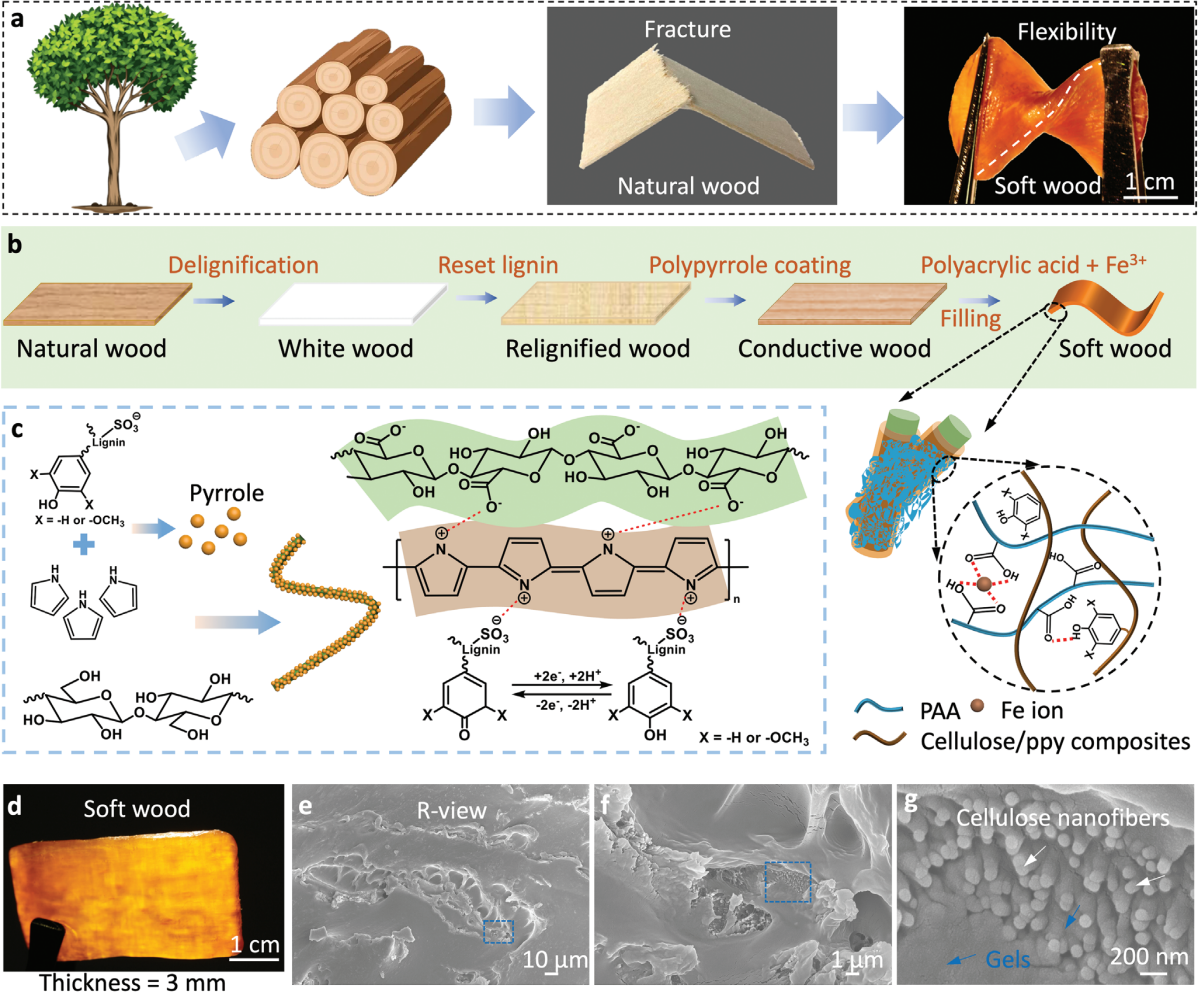
Design principle and characterization of wood‐framed hydrogels. a) A piece of soft wood prepared from tree. b) Key steps of conversing natural wood to soft wood. c) Formation principle of cellulose/polypyrrole composites by coating pyrrole on cellulose fibers. d) Photograph of soft wood. e–g) cascaded magnified scanning electron microscopy images of soft wood from the R‐view (radially cut wood, normal to its growth direction).

We prepare the soft and electrically conductive wood by first delignifying and resetting lignin, followed by filling wood nanochannels with conductive polypyrrole (PPy) and polyacrylic acid (PAA, **Figure** **1**b). Delignification entails treating balsa wood^[^ ^15^ ^]^ with a mixture of NaOH and Na_2_SO_3_ solutions for 6 h to remove lignin, yielding lignosulfonate. Further boiling the wood chips in the NaClO_2_ solution bleaches the wood to appear white (denoted as “white wood”). Next, we use TEMPO oxidation (24 h) to convert hydroxymethyl groups to carboxyl groups at the C6 position of cellulose glucose rings. It makes the wood highly water favorable and thereby facilitates the subsequent relignification by resetting lignosulfonate into the microchannels of white wood via hydrogen bonds between lignosulfonate and cellulose frame.^[^ ^16^ ^]^ Note that the directional porous structure of wood remains after delignification and relignification processes (Figure S1, Supporting Information), indicating a homogeneous lignin coating.^[^ ^14^, ^17^ ^]^

The natural woods are typical electrical insulators.^[^ ^18^ ^]^ To overcome this limit, we coat the internal channels of woods with conductive polymers.^[^ ^19^ ^]^ Hereinto, pyrrole monomers (diluted with 1,4‐dioxane) are used and polymerized within relignified woods (Figure 1c), wrapping up the cellulose frame of woods (denoted as “conductive wood” in Figure S1 (Supporting Information)). The homogeneous polypyrrole coating confers electrical conduction through woods, while preserving their directional porous structure.

To allow woods with flexible and resilient structures, filling their internal microchannels with gels is a plausible solution.^[^ ^14^, ^17^, ^20^ ^]^ Inspired by previous reports, where polyacrylamide gels are filled in wood,^[^ ^20b^ ^]^ acrylic acid monomer is used instead for a favorable affinity to polypyrrole via electrostatic interaction. In addition to initiator ammonium persulfate, the preparation involves Fe^3+^ ions. The dual role of Fe^3+^ ions are, 1) chelating strongly to phenolic hydroxyl and carboxyl groups in woods and gels, crosslinking the gel network,^[^ ^21^ ^]^ 2) enhancing ionic conductivity. After polymerization, the wood with ≈3 mm thickness is twistable without rupture (denoted as soft wood, Figure 1a,d) in contrast to pristine wood that is fractured upon twisting. Moreover, the wood is translucent because of close refractive indices between the wood frame (cellulose refractive index ≈ 1.53, lignin 1.61) and polyacrylic acid (refractive index = 1.53).^[^ ^22^ ^]^ Notably, the transmission at visible and infrared light regions of soft wood is improved by ≈20% compared to white wood (Figure S3, Supporting Information).

For a further understanding of soft wood formation, multi‐tools are used to characterize the samples. By Fourier‐transform infrared spectroscopy (Figure S4, Supporting Information), peaks at 1730 cm^−1^ corresponding to the C═O stretching vibration indicate the conversion of hydroxyl to carboxyl groups after the TEMPO modification. The soft wood features absorption peaks at 800 cm^−1^ for C─H bending, 1161 cm^−1^ for C─OH stretching of phenol in lignin molecules, 1234 cm^−1^ for C─N stretching of pyrrole, 1400–1450 cm^−1^ for C═C skeletal vibration, 1694 cm^−1^ for C═O stretching vibration of carboxyl in PAA, respectively, indicating the formation of wood‐based composite hydrogels.

In addition, X‐ray photoelectron spectroscopy is used to explore the valence state and functional groups in conductive and soft woods (Figure S5, Supporting Information). For conductive wood, the C1s spectrum shows peaks at 286.8, 283.9, and 282.4 eV corresponding to C═O peak in cellulose fibers, and C‐OH peak and ring in pyrrole, respectively (Figures S6a and S8a, Supporting Information. The N1s peaks at 400.7, 399.7, and 398.1 eV are associated with N═O, N─C aromatic, and N─C bonds, respectively (Figure S7a, Supporting Information). Transferring to soft wood significantly changes the characteristic peak intensity and the position of functional groups. For C1s scan, the peaks’ intensity of both C═O and C─O shows obvious enhancement due to the PAA addition and the formation of hydrogen bonding between the PAA, lignosulfonate and cellulose fibers (Figures S6b and S8b, Supporting Information). Compared to conductive wood, an increase in intensity of the N═O peak at 399.6 eV is observed in soft wood, while the nonaromatic N─C at 396.3 eV decreases sharply, hinting more hydrogen bonds are present (Figure S7b, Supporting Information). Furthermore, both the aromatic and nonaromatic N─C peaks are shifted due to the production of hydrogen bonds.

To observe the internal morphologies of soft wood, we perform cross‐section observations perpendicular (R‐view) and parallel (L‐view) to its grain orientation. Along the radial direction (R‐view), the micro/nanochannels of the wood are filled with the PAA gel (Figure 1e). A close inspection indicates that the cellulose fibers are wrapped fully by PAA gel (Figure 1f,g). Consistent results can be seen from the longitudinal view (L‐view, Figure S9a, Supporting Information). Furthermore, scanning electron microscopy with energy dispersive spectroscopy reveals the uniform distribution of C, O, N, and Fe elements within the softwood (Figure S10, Supporting Information).

### 2.2 Wood‐Framed Devices

**Figure 2:**
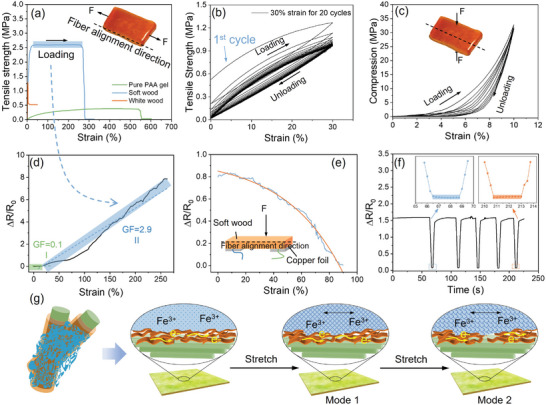
Characterization of soft wood. a) The tensile strength measurements of pure PAA gel, white wood, and soft wood. b) Tensile–strain curves of soft wood over 20 loading–unloading cycles up to 30% strain. c) Compressive stress‐strain curves of soft wood over 5 loading–unloading cycles up to 10% strain. d) Relative electrical resistance variation upon stretching. Gauge factors (GF) are calculated based on the regions highlighted with colored backgrounds. e) Relative electrical resistance variation of soft wood under pressure. The blue curve is the results, and the red line is the guide to eyes. f) Electrical response of soft wood to a mass (about 10 N of pressure). g) Schematic of dual‐mode electrical conduction within soft wood under stretching. Polypyrrole allows electronic conduction, while ferric ions induce ionic conduction. Upon stretching, polypyrrole is ruptured, leaving ionic conduction dominating.

Next, we characterize the mechanical properties of soft woods. The soft wood exhibits a combination of characteristic mechanical features absent in either white wood or pure PAA gel alone. Specifically, it displays a significantly higher tensile strength than pure PAA gel when stretched along the longitudinal direction but a comparable strength to white wood. Moreover, the soft wood shows a high strain, surpassing white wood while like pure PAA gel (**Figure** **2**a). This is ascribed to the robust wood frame and elastic PAA gel filler.

Indeed, every constitutive component in soft wood affects its mechanical property. As shown in Figure S12a, Supporting Information, the relationship between mass ratio of conductive wood to PAA and ultimate stress is noteworthy. With an equal ratio of 1:1, the highest stress of 2.7 MPa is attained, while as the ratio increases further, both stress and strain decrease. Moreover, the tensile‐strain curves show the transition from rigid to elastic behavior, where the 2:1 ratio is like that of white wood, while the 1:4 ratio resembles that of pure PAA gels. The adjustments in pyrrole concentration or TEMPO‐modification duration have minor impacts on tensile stress. But they do impact the strain levels, i.e., increasing the pyrrole concentration increases the strain (as seen in Figure S12b,c, Supporting Information). These variations could stem from the increased amount of hydrogen bonding among the modified cellulose frame, polypyrrole, and PAA.

Notably, this soft wood can withstand 20 cycles of stretching at 30% strain without discernible fracture (Figure 2b). The considerable hysteresis during the initial loading and unloading process might result from the reconstruction of affinity between Fe^3+^ ions, lignosulfonate, PAA chains, and cellulose fibers. During the cyclic testing, the gradual overlap of the stress–strain curves for soft wood at 5–50% strains suggests the enduring stability of soft wood under repetitive usage (Figure S13, Supporting Information). Nonetheless, there is noticeable energy dissipation once the strain reaches 100%. This arises from the irreversible rupture of covalent bonds. From these results, we conclude that reversible hydrogen bonds and metal‐coordination bonds contribute significantly to the elasticity of soft wood. In addition, the soft wood demonstrates exceptional compression resistance along the grain direction (Figure S14, Supporting Information). Under 5 cycles of 10% compressive strain, no visible displacement or breakage occurred, signifying appealing fatigue resistance (Figure 2c).

To explore the potential application of soft wood in handwriting electronics, we first prepare a sample with dimensions of 40 mm × 15 mm × 3 mm. We then evaluate its conductivity through various experiments. Specifically, the strain sensitivity of soft wood is measured by subjecting it to a stretching speed of 0.4 mm s^−1^. For this purpose, the gauge factor (GF) was introduced, defined as the ratio of relative resistance change (Δ*R/R*_0_) to the mechanical strain *ε*.^[^ ^23^ ^]^ Figure S15 (Supporting Information) shows the data collected upon shifting writing forces of fingers, resulting in varying resistance values. In Figure 2d, the relative resistance change increases with increasing tensile strain in two linearly responsive regimes: 0–20% with a GF of 0.1 and 20–250% with a GF of 2.9. The reason underly this trend lies in the manner that the soft wood exhibits two modes of electrical conduction. As illustrated in Figure 2g, the PPy and Fe^3+^ ions offer two types of electrical conduction, i.e., electronic and ionic conductions, respectively. At low strains, both modes operate concurrently, leading to proportional increase in resistance according to the strain (Mode 1). As the strain surpasses 20%, however, the network of PPy ruptures, resulting in significant resistance amplification while preserving the ionic conduction pathway (Mode 2). Moreover, we examine how soft wood responds to longitudinal compression. As depicted in Figure 2e, the resistance‐change decreased substantially with increasing the strain.

We next study the electrical stability of soft wood by repeating the compression/stretch‐release experiments. The relative resistance‐change decreases upon the application of compression and recovers once the compression is released (Figure S16, Supporting Information). This responsiveness is accurate and identical to the same pressure over multicycle of compression release (Figure 2f). Soft wood also exhibits exceptional long‐term stability during 500 consecutive cycles of stretch‐releasing at a strain of 20% (Figure S17, Supporting Information). Remarkably, it remains stable even under high levels of strain ranging from 5% to 100% (Figure S18, Supporting Information), indicating its suitability in writing pad. These results collectively suggests that soft woods are well suited for use in handwriting recognition systems.

### 2.3 Handwritten Information Collection and Manual Feature Extraction

**Figure 3:**
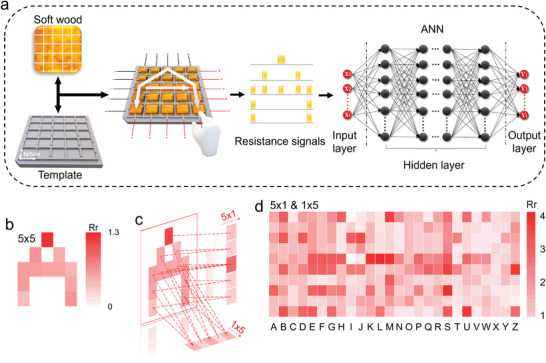
Working flow of handwritten alphabet recognition on signal collection from hydrogel arrays to data pre‐processing by machine learning (ML). a) Alphabet recognition via artificial neural network (ANN) training. Resistance signals collected from handwriting on an electrically connected 5 × 5 hydrogel array composed of soft wood, as the input for ANN training and output learned alphabet. b‐d) Data visualization and feature reduction & extraction. b) Letter A represented in the 5 × 5 array (*R*_r_ is the relative resistance relating to handwriting intensity). c) Feature extraction of the 5 × 5 array by mapping in rows (5 × 1) and columns (1 × 5). d) Alphabet represented by the combination of 5 × 1 & 1 × 5 (transposed) signal patterns.

To realize a prototype of a handwriting input signal collector, we devise soft woods with the same shape into an array with a 5 × 5‐pixel resolution. Upon writing, resistance signals are recorded and arranged into a 5 × 5 matrix according to the time sequence (**Figure** **3**a). Subsequently, these results are used to train the ML network, specifically with the ANN algorithm.^[^ ^10^, ^24^ ^]^ Handwritten letters often vary in terms of intensity, sequence strokes, and form, making them difficult to accurately recognize.^[^ ^25^ ^]^ Our approach involves recording spatially distributed data in a 5 × 5 matrix as input (Figure 3b) to eliminate the impact of stroke sequence on our results. Then we applied manual feature extraction (MFE) to minimize the influence of letter shapes by mapping the 5 × 5 matrix into 5 × 1 rows or 1 × 5 columns (Figure 3c), instead of the widely adopted automated feature extraction^[^ ^26^ ^]^ that excels for the big data or specific data type (e.g., spatial‐temporal data^[^ ^27^ ^]^), which might be problematic for the homogenous data with small volume^[^ ^28^ ^]^ in this case. Although the applied MFE can efficiently remove redundant information and improve data compatibility, but the other side of the coin with it may impair the accuracy of model.^[^ ^29^ ^]^ As shown in Figure S19 (Supporting Information), feature integration leads to pattern similarity for either 5 × 1 or 1 × 5 datasets, thereby confusing the processes of alphabet learning and recognition. Hence, we integrated 5 × 1 & 1 × 5 datasets into one set (Figure 3d), effectively improving the alphabet discrimination while simultaneously reducing the feature's dimensionality from 25 (5 × 5) to 10 (5 × 1 & 1 × 5). For subsequent model construction, we used three datasets (5 × 1, 5 × 1 & 1 × 5, and 5 × 5) to compare and evaluate the performance of MFE. Classification characteristics of three datasets are examined beforehand (Figure S20, Supporting Information) with the overall quality: 5 × 5 > 5 × 1 & 1 × 5 > 5 × 1.

### 2.4 Modeling and Evaluation

**Figure 4:**
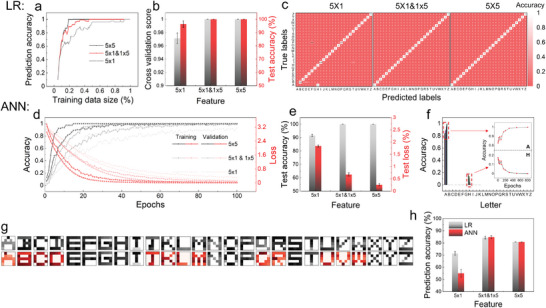
Learning results using logistic regression (LR) and ANN algorithms, training & predicting on datasets of 5 × 1, 5 × 1 & 1 × 5 and 5 × 5. a–c) LR results. a) Prediction accuracy of models built by varying the size of training data. b) Model evaluation on cross‐validation score and test accuracy of 20% dataset. Error bars represent the five times of random split of dataset into training (75–85%) and testing (15–25%) groups. c) Visualized predictive results for each letter. d–f) ANN results. d) Model fitting performance on 5 × 1, 5 × 1 & 1 × 5 and 5 × 5, evaluated by accuracy and loss on training and validation datasets. e) Prediction performance on the test dataset. Error bars indicate the hyperparameters tuning (5 combinations). f) Learning process of letter “A,” accuracy increases (possibility of A) with increasing epochs (training times). g) Visualization of training (1^st^ row) and new test (2^nd^ row, half changed) datasets. h) Overall models’ prediction accuracy on the new test dataset (g). The error bars reflect both random split of dataset and hyperparameters tuning.

To evaluate the selection of algorithms, we explored the logistic regression (LR) and ANN as the representatives for the traditional (low‐complexity) and advanced (high‐complexity) algorithms, regarding to their efficiency and model accuracy. LR can achieve high accuracy with relatively low computational cost (**Figure** **4**a–c). Specifically, up to 50% of training data is sufficient to reach a 95% prediction accuracy across all three datasets, with the best performance using only 20% of the 5 × 5 dataset (see Figure 4a). Cross‐validation score and test accuracy (on 20% dataset) are also consistently above 95%, demonstrating exceptional fitting performance (Figure 4b). Furthermore, the prediction error distributions in Figure 4c (from 5 × 1 to 5 × 1 & 1 × 5), suggest that appropriate selection of feature extraction enables effective information integration and better predictions. In contrast, either 5 × 1 or 1 × 5 fails to distinguish some certain letters’ signal, e.g., “A&H” and “H”&’M’&’N’&’U’&’W’ (Figure S19, Supporting Information). But the combination of them results in unparalleled signal patterns of the alphabet (Figure 3d), thus facilitating the ML modeling.

In contrast, ANN often performs better than LR at handling big data with advanced adaptability and accuracy.^[^ ^30^ ^]^ Figure 4d–f demonstrates the process and evaluation of the ANN model. As shown in Figure 4d, 75% of data are used for training and validation, the fitting process of 5 × 1, 5 × 1 & 1 × 5 and 5 × 5 are recorded and evaluated with increasing epochs (learning times), which are all well‐fitted with the enhanced performance with the increasing features. In Figure 4e, it indicates 5 × 1 & 1 × 5 outperforms 5 × 1, with the 100% prediction accuracy—an indicative of potential model overfitting.^[^ ^31^ ^]^ Continuously, the observed optimization of 5 × 5, indicated by the decreasing test loss, further validates the on‐going model overfitting. In conclusion, the observation collectively manifests that the choice of 5 × 1 & 1 × 5 proves to best improve the model accuracy while mitigating the risk of overfitting among the feature groups. Besides, benefitting from the ANN model, monitoring output‐change relating to model adjustment facilitates model optimization, and better visualizes the learning process.^[^ ^32^ ^]^ For example, when studying the letter “A” in 5 × 1 patterns (Figure 4f), the output consists of 26 probabilities distributed from “A” to “Z.” A proportion is wrongly assigned to “H” and gradually re‐assigned to “A” with increasing learning cycles (Figure 4f, inset). Demonstration of the letter “B”–“Z” is given in Figure S20 (Supporting Information). Additionally, the result of K‐means clustering suggests appropriate feature extraction could better reserve the data quality in their distinctiveness, i.e., 5 × 1 & 1 × 5 feature type outperforms 5 × 1 (Figure S21, Supporting Information).

Limited data resource poses significant challenges for ML applications at the laboratory level, leading to suboptimal fitted models and poor predictions.^[^ ^33^ ^]^ In this study, we exploratively collected data solely from one individual's handwritings. Whereas, to evaluate the generalization ability of the model, we check the prediction performance of the overall built models with LR and ANN on a new test dataset, in which half of the letters were designed clearly different from those of training data (Figure 4g). Figure 4h shows the prediction accuracies for 5 × 1, 5 × 1 & 1 × 5, and 5 × 5 are 71.4, 84.4, and 80.8% respectively, demonstrating the optimal generalization ability of 5 × 1 & 1 × 5. Further complementary analysis on LR models (Figure S22, Supporting Information) validated the utmost effectiveness of 5 × 1 & 1 × 5, where 5 × 1 proved to be underfitted and 5 × 5 was overfitted as indicated in Figure S22b, Supporting Information. For both algorithms, 5 × 1 & 1 × 5 performs the best, suggesting the appropriate MFE could efficiently enhance the model generalization ability. As complementary, we also compared the prediction performances between 5 × 1 & 1 × 5 and principal component analysis (PCA), the latter is a common ML technique of automated feature extraction. For the classification task on the training and testing data shown in Figure 4g, the prediciton accuracy of 5 × 1 & 1 × 5 is overall higher than PCA., i.e., 4.85% accuracy enhancement for LR and 10.00% for ANN (Figure S23, Supporting Information), which demonstrates the optimal MFE could be a better choice than automated ML approach for small‐data tasks, with improved model interpretability and prediction accuracy.

### 2.5 Phrase Recognition

**Figure 5:**
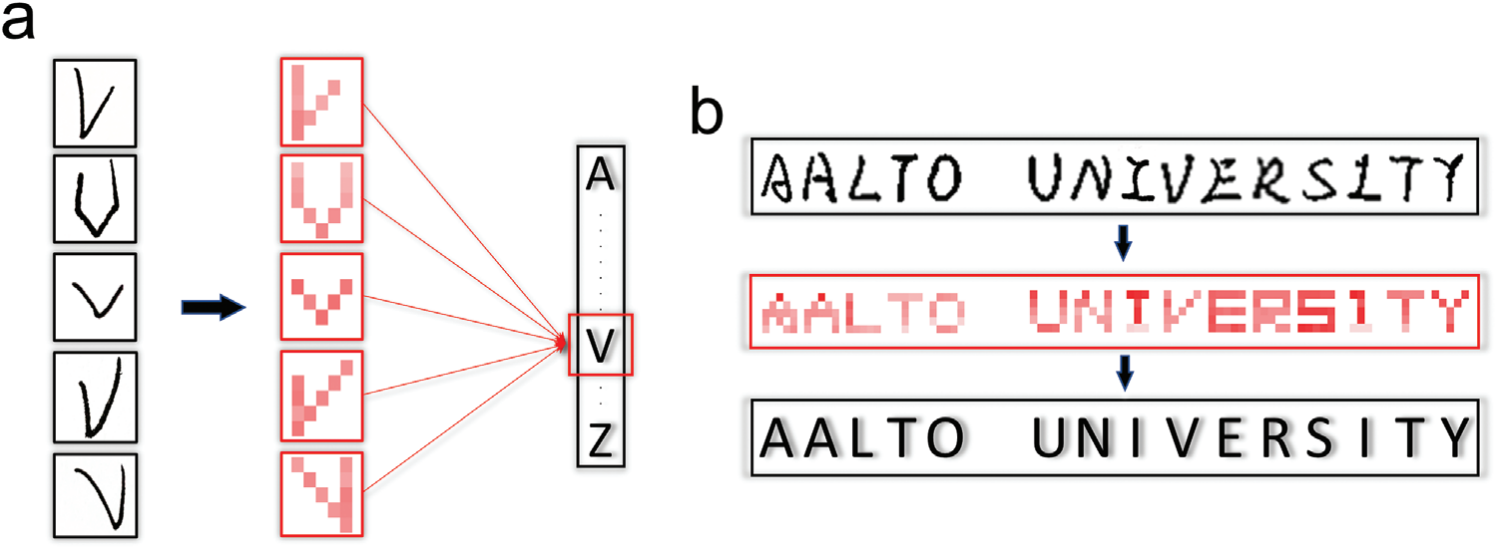
Demonstration of model generalization and phrase recognition. a) Recognition on five untrained letters “V” with 80% accuracy. b) Handwritten phrase recognition of “AALTO UNIVERSITY” with 93% accuracy.

Further practical demonstration based on alphabet learning verified the model robustness. As visually depicted in **Figure** **5**, the transition from handwriting to synchronized resistance signal recording has extracted key features, showcasing the transformation from handwriting to digitalized form. Then with the model built on the combination of ANN and 5 × 5 feature type, the prediction on 5 groups of untrained handwritten letters “V” achieves an accuracy of 80% (Figure 5a). Moreover, for handwritten long phrases “AALTO UNIVERSITY,” with the model built on LR and 5 × 1&1 × 5 feature, the prediction accuracy could reach up to 93% (Figure 5b). It needs to be pointed out those predictions are made based on a handful of training data, to demonstrate the effectiveness of the MFE‐directed ML networks with reduced complexity.

## 3 Discussions

Woods, a renewable resource, have been utilized as the starting materials for fabricating “green” flexible electronics capable of dual conductivity modes, serving as touchpads for handwriting recognition assisted by ML. To achieve this, we first demonstrated an extraction‐refill strategy to prepare highly flexible and electrically conductive haptic sensors based on wood. Then, these sensors were manufactured into arrays with a 5 × 5 resolution and integrated into a circuit for collecting handwriting input. By employing ML techniques with effective MFE and simplified algorithmic structure, we thoroughly evaluated the performances of different ML networks. Detailed outputs were presented, and the generalization ability of optimal models was assessed. Inspiringly, emphasizing on data‐preprocessing other than algorithms facilitates model optimization, making it appealing to experimentalists (Table S2, Supporting Information), particularly in the domain of handwriting recognition and their applications into wearable devices. Moreover, our work promotes the design of “tiny ML”^[^ ^34^ ^]^ by simplifying the model structure, and envisions the promising integration of MFE with automated feature extraction to help improve model interpretability and prediction performance, to meet the increasing computing demand of advanced applications (e.g., healthcare, mobile and wearable devices). In summary, this work showcases the fabrication of wood‐framed haptic sensor arrays, enabling the recognition of alphabet handwriting through ML encoding. It promotes sustainability and public participation in the design of integrated systems powered by embedded ML.^[^ ^35^ ^]^ As an onset, this work holds promising prospects for further development in sustainable healthcare, smart homes, and digital sports.

## 4 Experimental Section

### Materials and Chemical Reagents

Balsa wood was bought from Jufashion Co. Ltd., China, and cut to the dimensions of 1 × 20 × 60 mm^3^. Sodium hydroxide (NaOH), sodium sulfite (Na_2_SO_3_) (analytical grade, Sigma‐Aldrich), hydrogen peroxide solution (H_2_O_2_, 30 wt%), 3,4‐ethylenedioxythiophene (EDOT, 99%, Acros Organics), pyrrole, sodium persulfate (Na_2_S_2_O_8_), iron(III) chloride (FeCl_3_), acrylic acid (AA), and ammonium persulfate (APS) were used as received. Deionized (DI) water was used for the preparation of all solutions.

### Fabrication of Wood‐Based Hydrogel—Bleaching the Natural Wood

Basswood was cut along or perpendicular to the growth direction with 1 × 50 × 50 mm (thickness, length, and width). The wood was boiled in a 2.5 m NaOH and 0.4 m Na_2_SO_3_ solution for 6 h. Then, the wood slices were separated from the brown mixture and were transferred to the boiling NaClO_2_ solution until they became sufficiently white (denoted as “white wood”). The white wood was then rinsed repeatedly in ethanol and deionized water to remove the residual chemicals. The as‐synthesized brown mixture was dialyzed with deionized water for 48 h and freeze‐dried, yielding brown powder (denoted as “lignosulfonates”).

### Fabrication of Wood‐Based Hydrogel—TEMPO Modification

The cellulose modification solution was prepared by mixing chemicals of 0.016 g TEMPO, 0.1 g NaBr and 3 mL NaClO aq. The wood samples were immersed thoroughly in the solution at room temperature for 5 h, accompanied with ultrasound for 30 min at 1 h intervals. Then, the wood samples were immersed into ethanol to stop reaction, and then washed by deionized water to pH 7.0. The freeze‐dried sample at −80 °C was denoted as “TEMPO‐modified white wood.”

### Fabrication of Wood‐Based Hydrogel—Synthesis of Conductive Wood

For PPy coating, the white woods and 1 mL of pyrrole monomer were sealed in a beaker at 2–8 °C for 7 d, respectively. Then, the wood samples were immersed in a mixture of lignosulfonates solution including Na_2_S_2_O_8_ and FeCl_3_. The mixture stayed at room temperature for 8 h and the polymerization was carried out. Finally, the obtained wood samples were freeze‐dried again for 24 h.

### Fabrication of Wood‐Based Hydrogel–Preparation of Soft Wood

Acrylic acid (AA), ammonium persulfate (APS), and ferric chloride (FeCl_3_) were added in a breaker placed in an ice bath. The soft wood was obtained after heating the mixture with conductive woods at 75 °C for 30 min. In contrast, the control groups with different lignin content and pyrrole content were prepared following the same procedures. The detailed formulation of each sample is listed in Table S2 (Supporting Information).

### Characterization

The functional groups of wood samples were recorded by PerkinElmer FT‐IR with ATR. The microstructure images of samples were monitored using a scanning electron microscope (FE‐SEM, Sigma VP, Zeiss, OtaNano Nanomicroscopy Center) observing an SE2 pattern and 15 kV. The tensile strength of samples was performed by a Instron 5567 materials testing system at room temperature. Olive oil applying to the surface of soft wood was applied to reduce the loss of water, and then tested at 1 cm min^−1^ stretching speed. The sensing test was performed by an LCR meter (TH2829A, Tonghui, China).

### ML Networks

Two algorithms (LR and ANN) and three types of datasets (feature types of 5 × 1, 5 × 1&1 × 5, and 5 × 5) were used throughout the model construction. The resistance data are recorded by constantly pressing the wood gel with a finger. The total samples are 520 with the 75% for training and the 25% for testing. Accordingly, the overall datapoints used in different model network are 2600, 5200 and 13 000, corresponding to three feature types of 5 × 1, 5 × 1 & 1 × 5, and 5 × 5. For the letter “V” recognition, there are 5 distinct inputs (Figure 5a) with each 5 repeating measurements, 25 testing samples in total. For the phrase recognition of “AALTO UNIVERSITY,” 15 samples (letters) were used as the input. The test models were built on ANN with 5×5 and LR with 5 × 1 & 1 × 5 feature types for above two recognitions. Besides, K‐means clustering algorithm was applied to evaluate the data quality for classification task, for the three feature types of 5 × 1, 5 × 1 & 1 × 5, and 5 × 5.

## Conflict of Interest

The authors declare no conflict of interest.

## Author Contributions

G.Y. and X.H. contributed equally to this work. G.Y., X.H., and B.P. conceived the conceptualization. G.Y. fabricated and characterized the wood‐based sensor arrays. X.H. designed the machine learning and handwriting recognition. G.Y. and X.H. analyzed the data with assistance from Z.M., G.Y., X.H., and B.P. wrote the manuscript with contributions from all authors.

## Supporting information

Supporting Information

## Data Availability

The data that support the findings of this study are available from the corresponding author upon reasonable request.
