# Supplementary material for: Alphabet Handwriting Recognition: From Wood‐Framed Hydrogel Arrays Design to Machine Learning Decoding
Source: Adv Sci (Weinh). 2024 Nov 4;11(47):2404437. doi: 10.1002/advs.202404437 (PMC11653617; doi:10.1002/advs.202404437)
Supplement: Supplementary file 1 — Supporting Information [file ADVS-11-2404437-s001.pdf]

ADVANCED SCIENCE
Open Access

# Supporting Information

for *Adv. Sci.*, DOI 10.1002/advs.202404437

Alphabet Handwriting Recognition: From Wood-Framed Hydrogel Arrays Design to Machine Learning Decoding

*Guihua Yan, Xichen Hu, Ziyue Miao, Yongde Liu, Xianhai Zeng, Lu Lin, Olli Ikkala and Bo Peng**

# Supplementary Information

## Alphabet handwriting recognition: from wood-framed hydrogel arrays design to machine learning decoding

*Guihua Yan,[1,2,3,5] Xichen Hu,[2,4,5] Ziyue Miao,[2,4] Yongde Liu,[1] Xianhai Zeng,[3] Lu Lin,[3] Olli Ikkala,[2] Bo Peng[2,4]**

[1] College of Environmental Engineering, Henan University of Technology, Zhengzhou 450001, China

[2] Department of Applied Physics, Aalto University, FI-00076 Aalto, Finland

[3] College of Energy, Xiamen University, 361102, China

[4] Department of Materials Science, Advanced Coatings Research Center of Ministry of Education of China, Fudan University, Shanghai 200433, China

[5] These authors contribute equally.

E-mails: pengbo006@gmail.com; peng_bo@fudan.edu.cn

**Table S1.** Composition details of various wood-derived hydrogels.

| Hydrogels | Lignin/all-wood (wt%) | Pyrrole (g) | White wood (g) | Water (mL) |
|---|---|---|---|---|
| Soft Wood | 8.7 | **0.2** | 0.2 | 10 |
| | 8.7 | **0.5** | 0.2 | 10 |
| | 8.7 | **1.0** | 0.2 | 10 |
| | 8.7 | **1.5** | 0.2 | 10 |
| | **3.9** | 1.0 | 0.2 | 10 |
| | **8.7** | 1.0 | 0.2 | 10 |
| | **12.1** | 1.0 | 0.2 | 10 |
| White wood | 0 | 1.0 | 0.2 | 10 |
| Control Hydrogel | 0 | | 0 | 10 |

**Table S2.** Qualitative comparison of built models

| Model | Model complexity | Computing cost | Model flexibility | Interpretability | Predictability | Generalization ability |
|---|---|---|---|---|---|---|
| I | ★ | ★ | ★ | ★★★ | ★★ | ★ |
| II | ★ | ★ | ★ | ★★★ | ★★★ | ★★★ |
| III | ★ | ★★ | ★ | ★★ | ★★★ | ★★ |
| IV | ★★★ | ★★ | ★★★ | ★★★ | ★★ | ★ |
| V | ★★★ | ★★ | ★★★ | ★★ | ★★★ | ★★★ |
| VI | ★★★ | ★★★ | ★★★ | ★★ | ★★★ | ★★ |

LR (**I**: 5×1, **II**: 5×1&1×5, **III**: 5×5), ANN (**IV**: 5×1, **V**: 5×1&1×5, **VI**: 5×5)

Six models were built based on two algorithms (LR and ANN) and three datasets (5×1, 5×1&1×5, and 5×5). The scoring criterion focuses on the comprehensive qualities of the models, and the score itself is an approximate estimate. Model II and V perform better than the others in terms of their performances, i.e., predictability and generalization ability. And models I-III differ from IV-VI primarily owing to the intrinsic algorithmic difference.

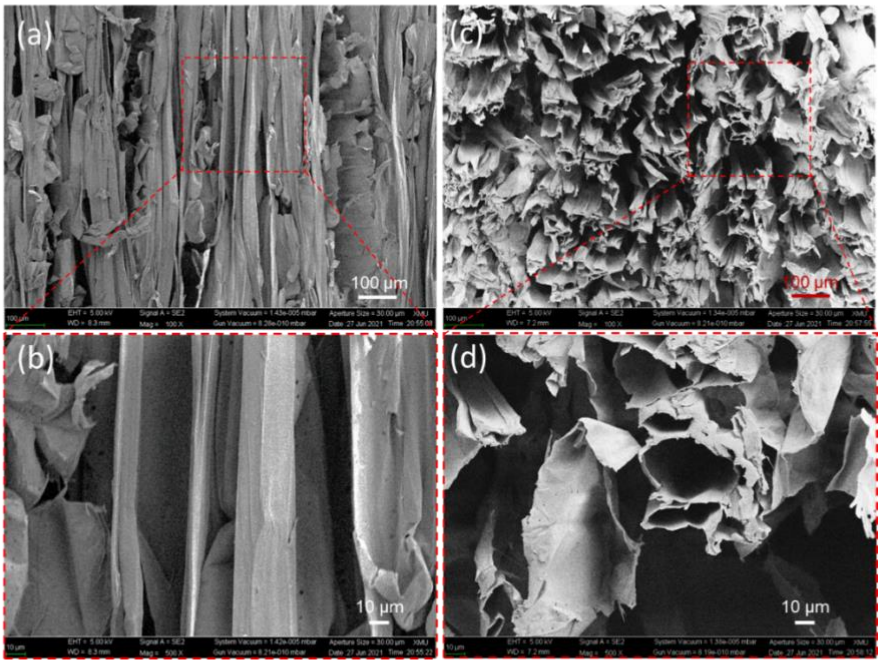


**Figure S1.** The porous structure of white wood.

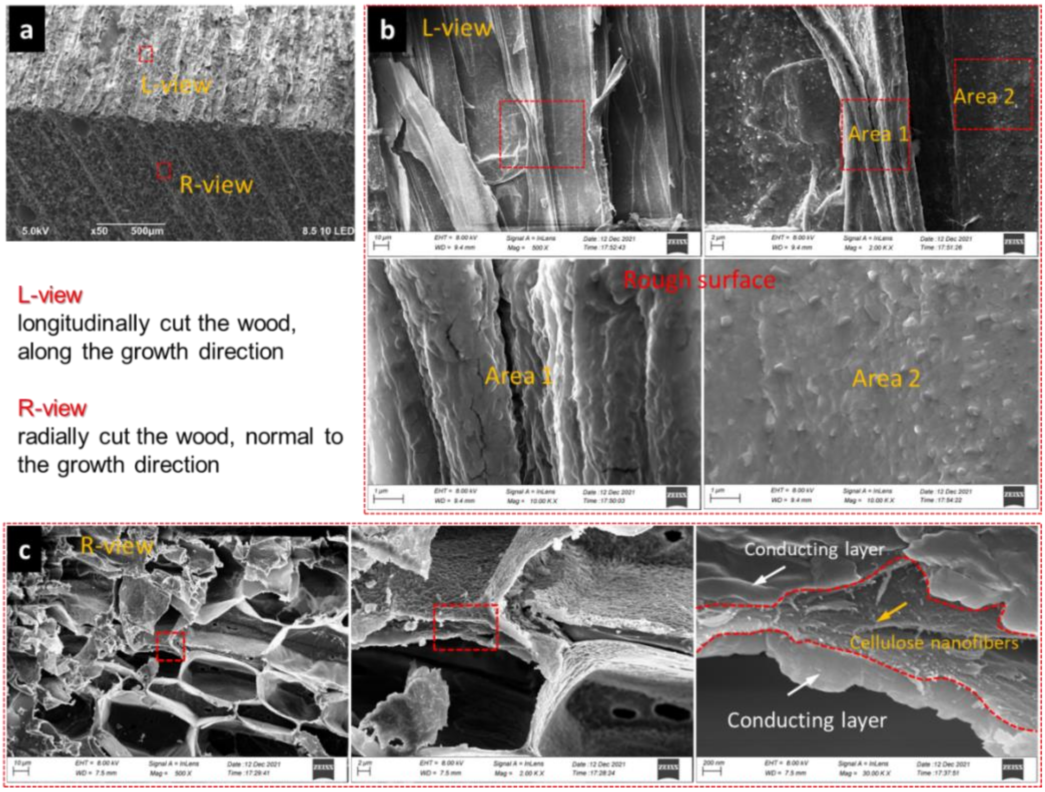


**Figure S2.** SEM images of conductive wood. a) the 3D image of the conductive wood. b) the side-view image of natural wood. c) SEM images of soft wood: the top-view SEM image and its magnified image.

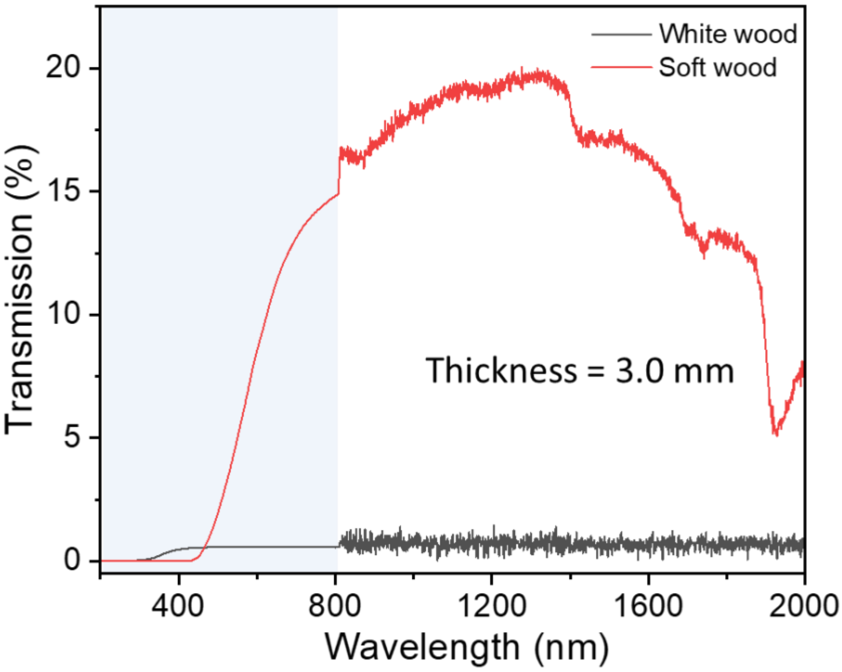


**Figure S3.** The transmission of the white wood and soft wood.

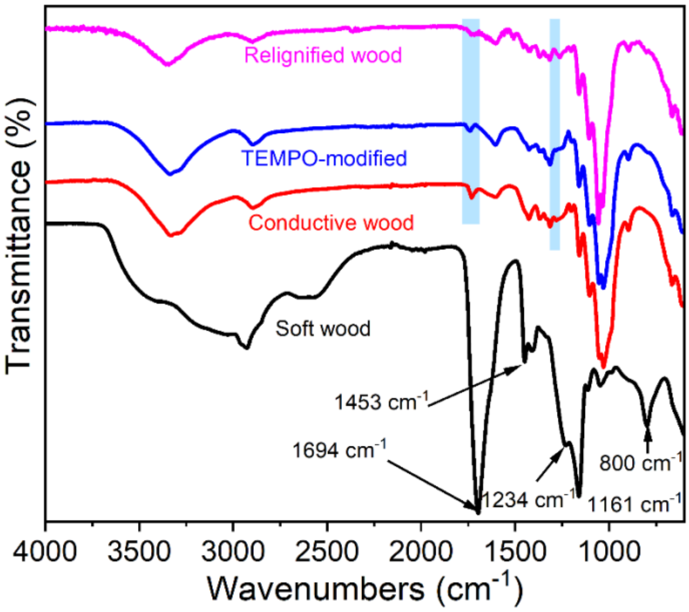


**Figure S4.** FTIR spectra of the relignified, TEMPO-modified, conductive, and soft woods.

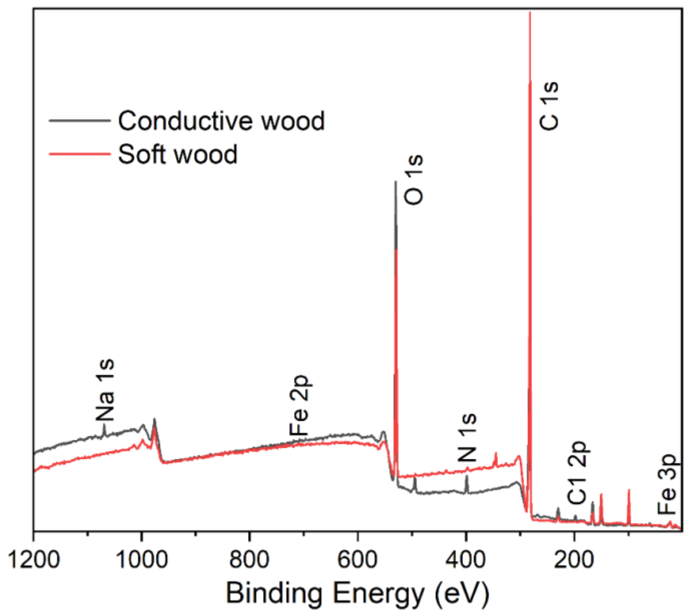


**Figure S5.** The XPS survey spectra of conductive and soft woods.

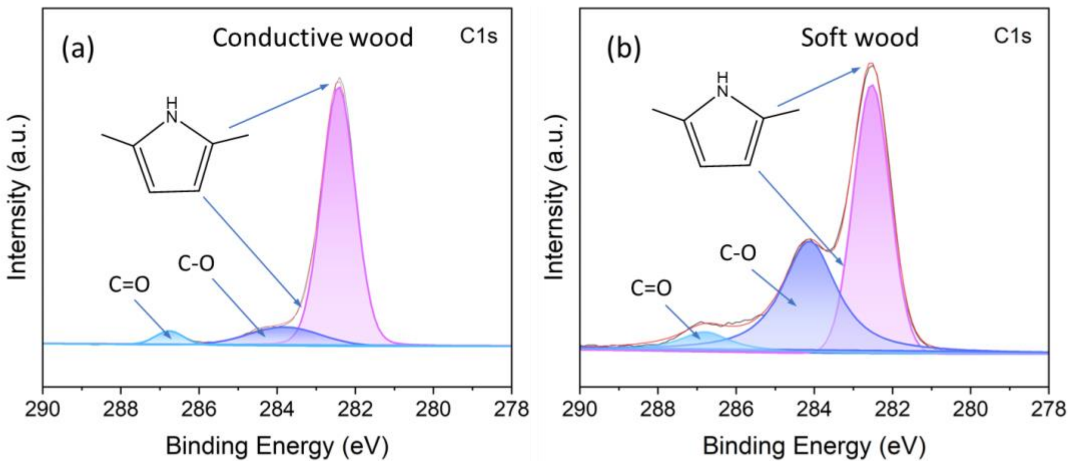


**Figure S6.** The C1s spectra of conductive and soft woods.

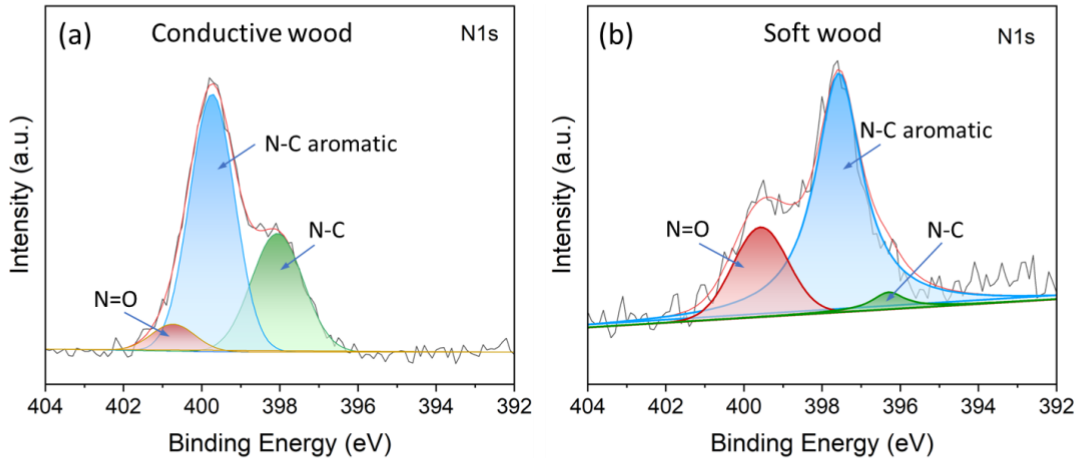


**Figure S7.** The N1s spectra of conductive and soft woods.

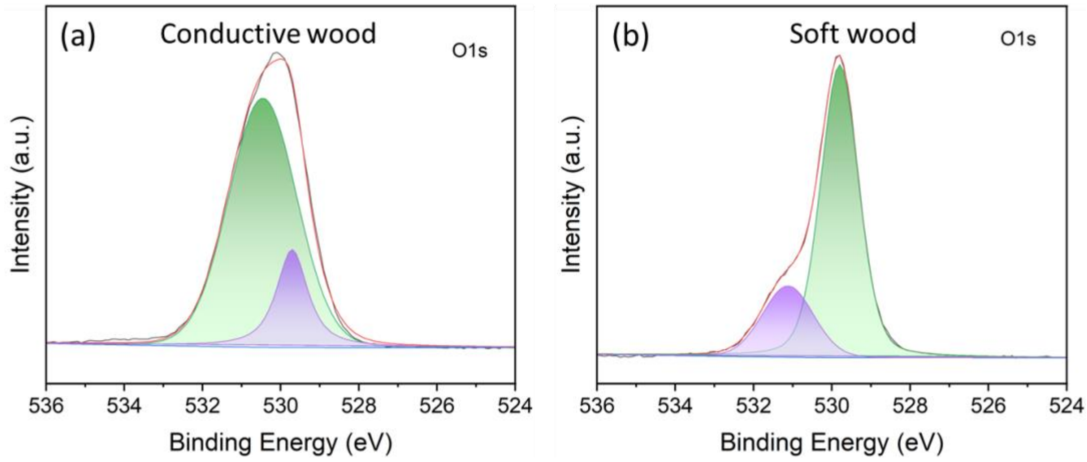


**Figure S8.** The O1s spectra of conductive and soft woods.

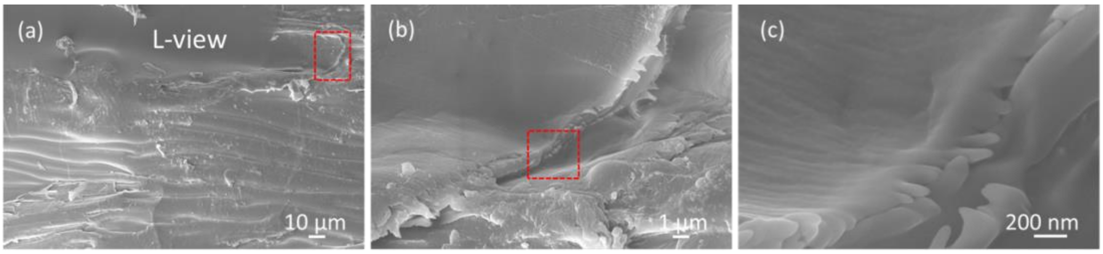


**Figure S9.** the side-view SEM image of soft wood.

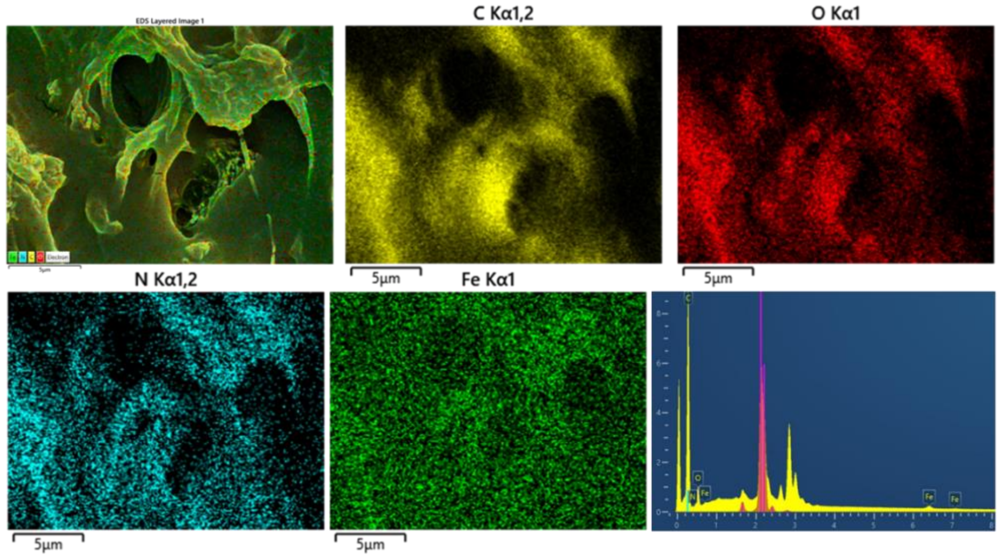


**Figure S10.** EDS-SEM images of soft wood.

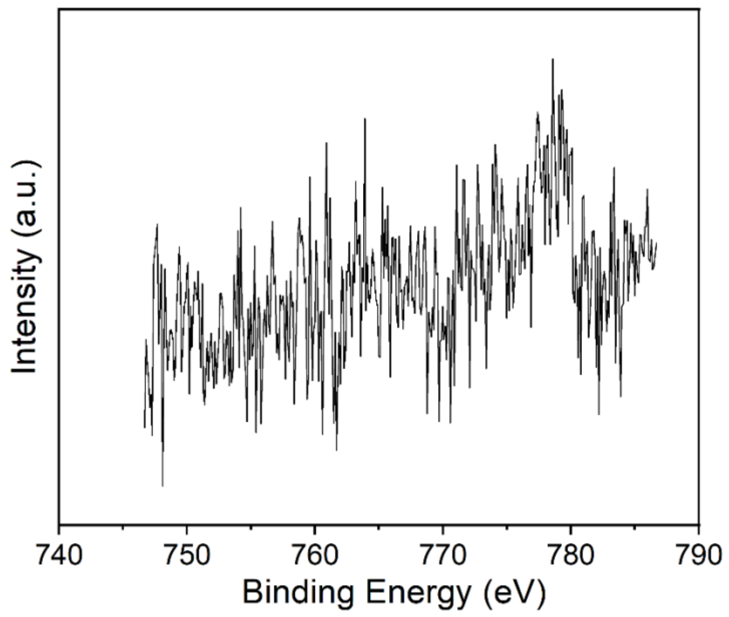


**Figure S11.** The Fe2p spectrum of soft wood.

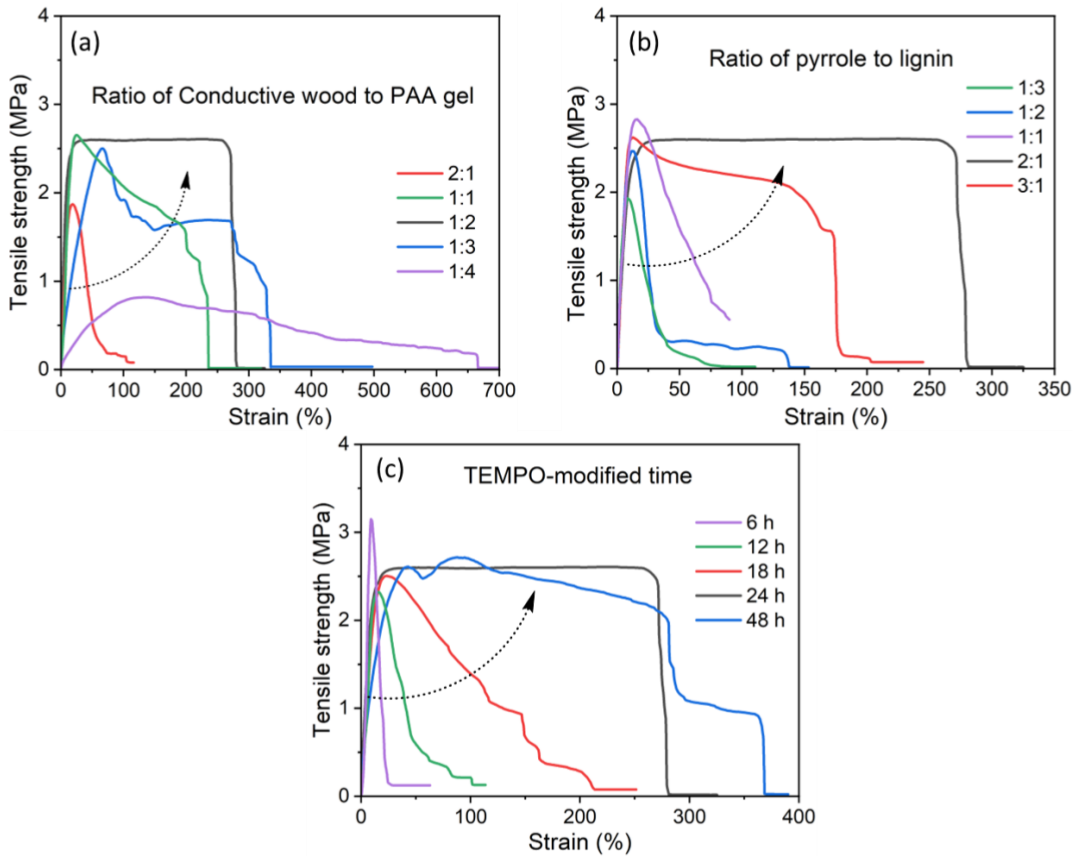


**Figure S12.** The effects of a) PAA content, b) pyrrole content, and c) TEMPO-modification time on the tensile strength of the as-prepared samples.

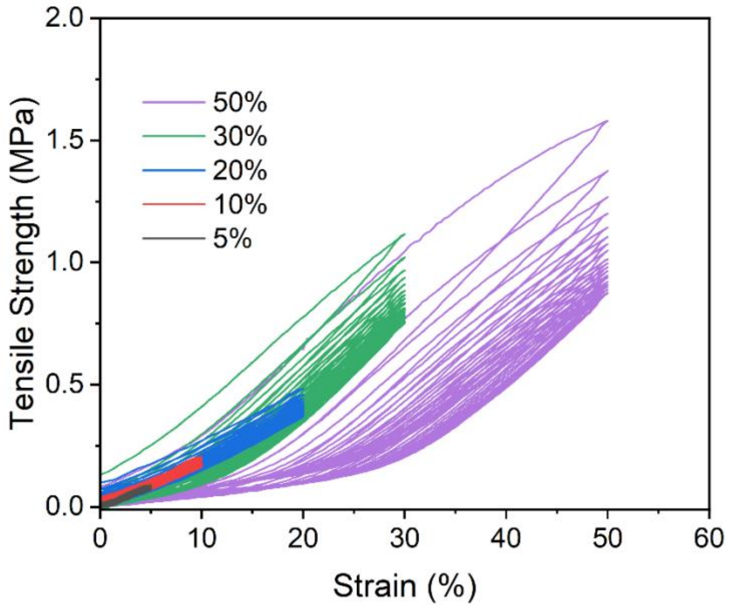


**Figure S13.** The cyclic tensile strength measurement of soft wood at 5, 10, 20, 30,

and 50% strain over 20 cycles.

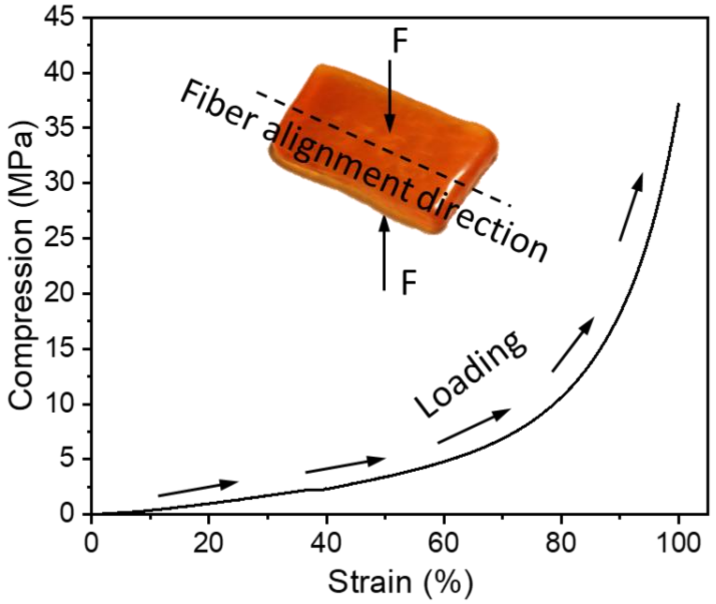


**Figure S14.** Compressive stress-strain characterization of soft wood.

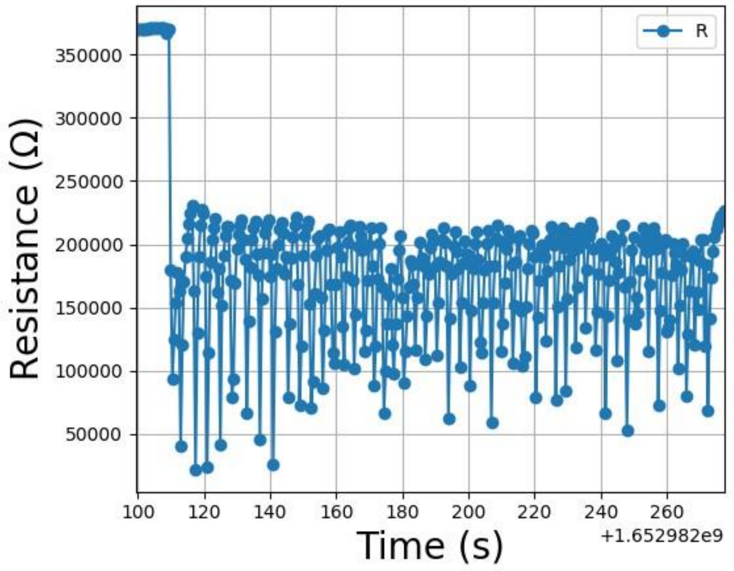


**Figure S15**. Resistance data of conducting wood gels that were pressed at intervals upon shifting writing forces of fingers.

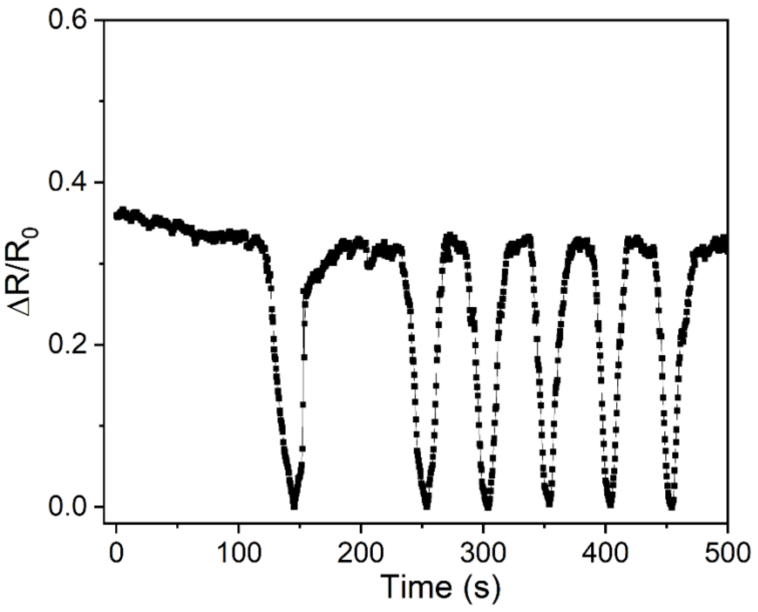


**Figure S16.** Relative resistance-changes of soft wood under quick compression-release.

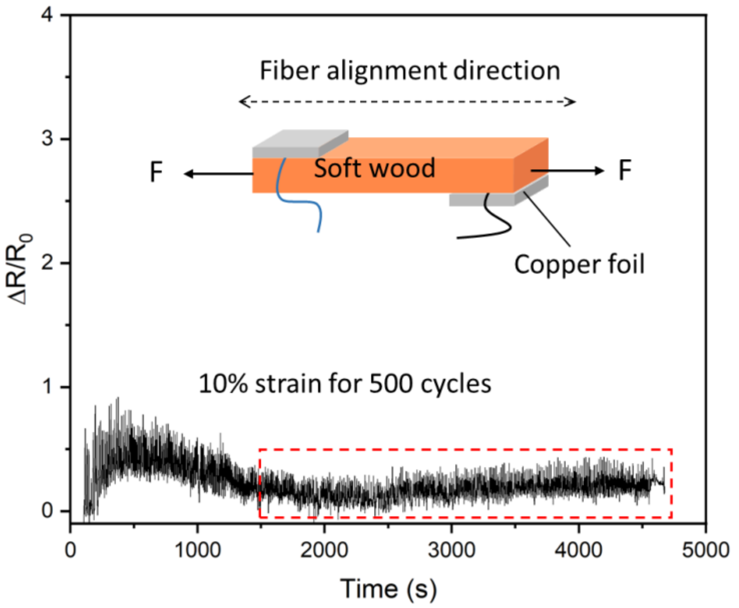


**Figure S17.** Long-term stability during 500 stretching-releasing cycles under a strain of 20%.

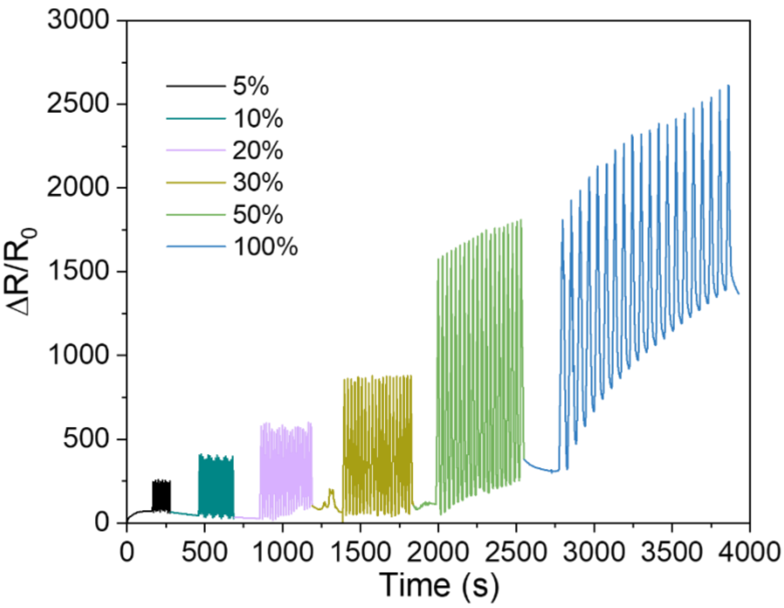


**Figure S18.** Relative resistance-change at the compressive strain of 5, 10, 20, 30, 50, and 100%.

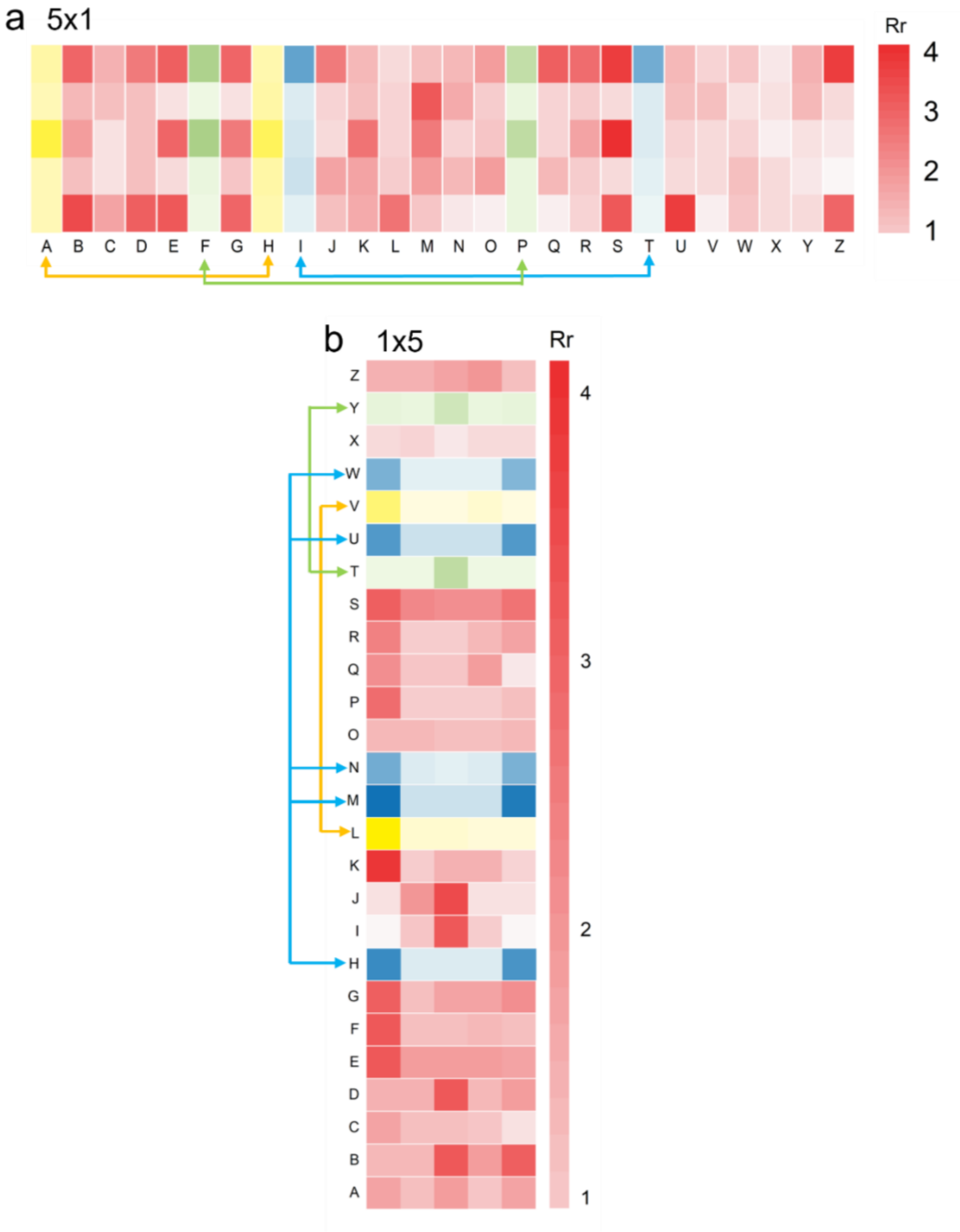


**Figure S19.** Feature reduction with undistinguishable letter patterns. a) alphabet represented in 5×1 signal pattern, mapped in rows from 5×5 matrix. Letters with same color (other than red) share similar 5 × 1 pattern, e.g., ‘A’&’H’ in yellow, ‘F’&’P’ in green, and ‘I’&’T’ in blue. b**)** alphabet represented in 1×5 signal pattern, mapped in columns from 5×5 matrix. Letters with the same color (other than red) share similar

1×5 pattern, e.g., ‘L’&’V’ in yellow, ‘T’&’Y’ in green, and ‘H’&’M’&’N’&’U’&’W’ in blue. Similar patterns indicate those letters can’t be well-distinguished by the ML network.

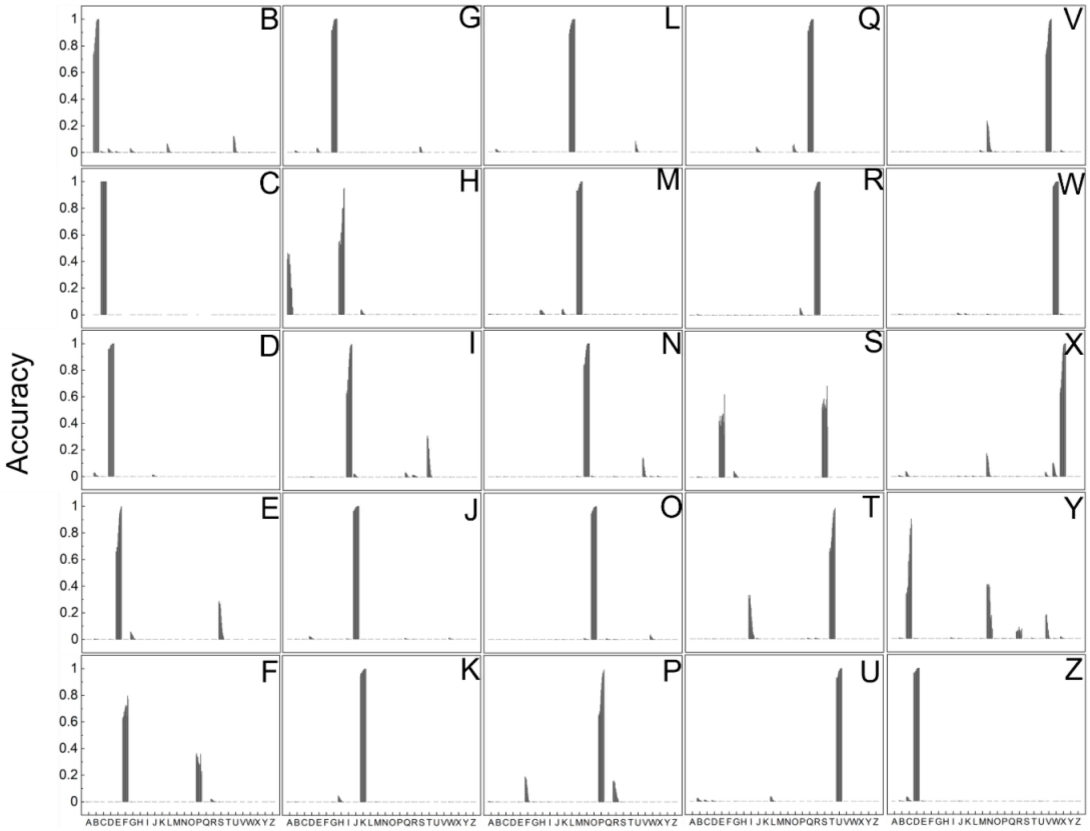


**Figure S20.** Visualizing the learning processes from ‘B’ to ‘Z’.

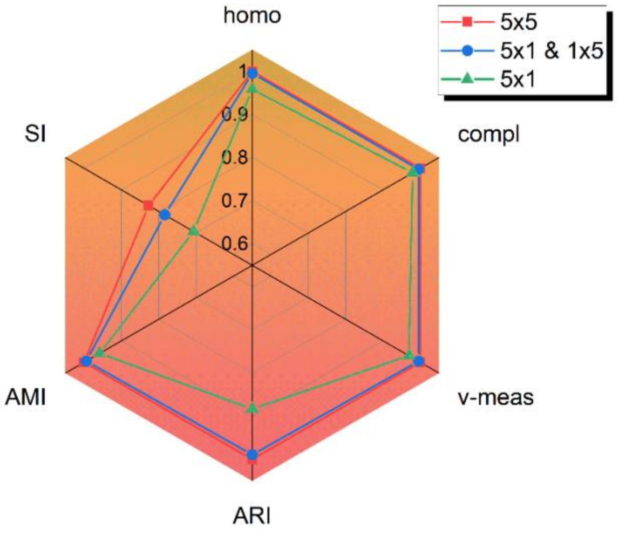


**Figure S21.** Data quality examination on three datasets of 5×5, 5×1&1×5 and 5×1 by using K-means clustering. ARI (Adjusted Rand Index), AMI (Adjusted Mutual Information), v-meas (v measure), compl (Completeness) and homo (Homogeneity) are performance metrics mathematically defined with score ([0-1], data quality increases with higher score) to evaluate data spatial distribution, scoring higher indicates better clustering quality, meaning each category (in this case is each letter) is better separated and more distinguishable. SI (silhouette coefficient) provides comprehensive measure on datapoints' distance, resembling the function of previous 5 metrics with score range of [-1,1].

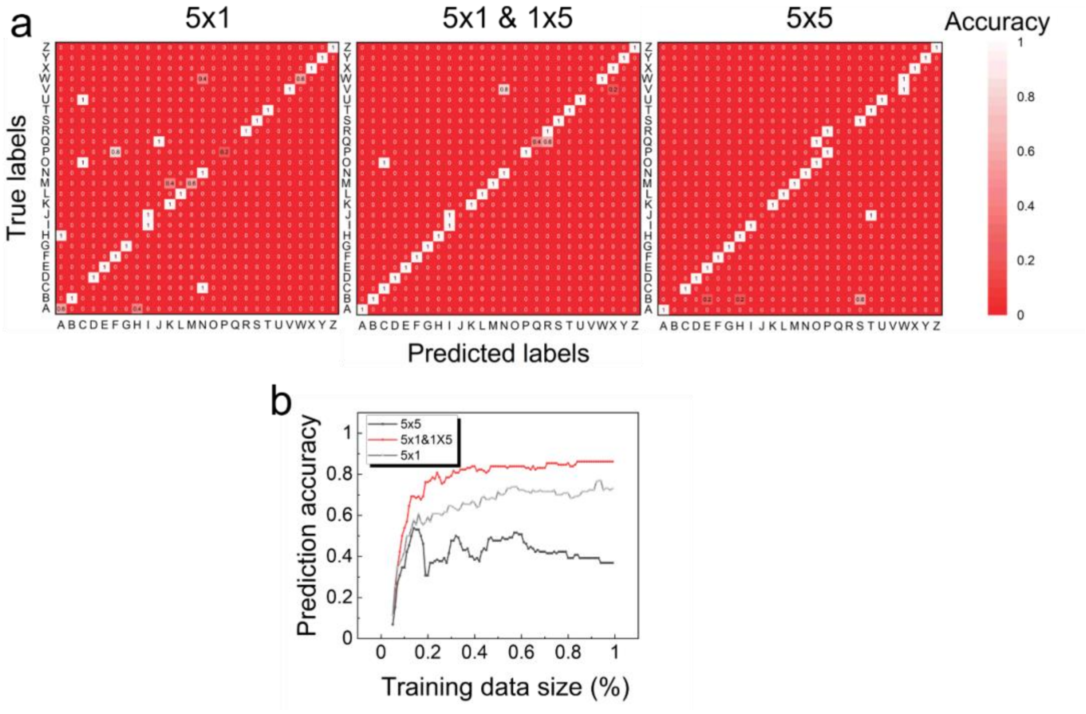


**Figure S22.** Predictive result distribution of models built on 3 datasets of 5×1, 5×1&1×5, and 5×5 using LR algorithm, test dataset is shown as in Figure 4g. a) Predictive results for each letter, showing probability distribution of prediction accuracy in heatmap. b) Prediction accuracy of models built on varying size of training data. Overall prediction performance of three datasets: 5×1&1×5 > 5×5 > 5×1.

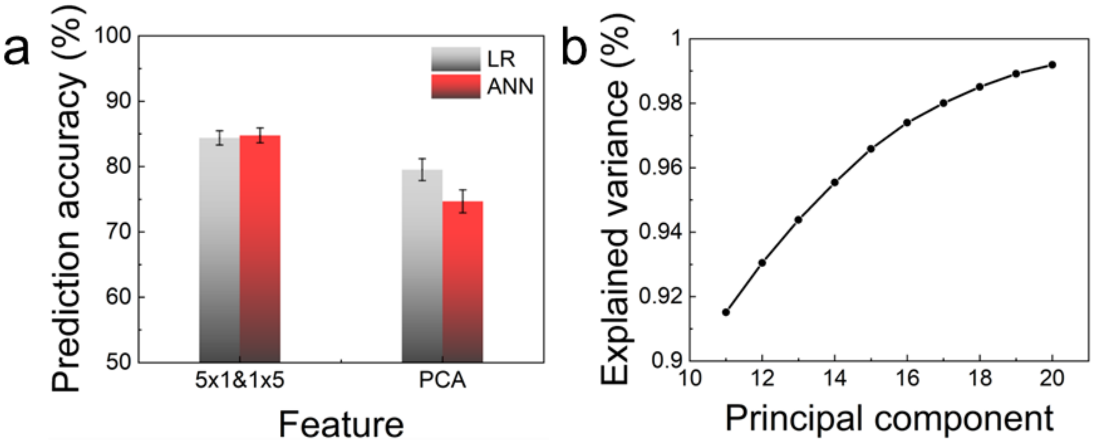


**Figure S23.** Comparison between MFE (5x1&1x5) and principal component analysis (PCA) on the model generalization ability. a) The prediction accuracy of models built on two algorithms, (i.e., LR and ANN) with two types of feature extraction (i.e., MFE (5x1&1x5) and automated feature extraction - PCA), on the test data shown in Figure 4g. b) Extracted features (equivalent to the number of principal components) via PCA corresponding to the reserved data information (equivalent to the explained variance). The error source of PCA in a) comes from the varying selection of principal components.
